# Congenital absence of the left atrial appendage: a positive coincidence for the electrophysiologist? A case report

**DOI:** 10.1093/ehjcr/ytae653

**Published:** 2024-12-07

**Authors:** João Gabriel Batista Lage, Rafael Augusto Lethier Rangel, Nilson Araújo de Oliveira Júnior, Martha Valeria Tavares Pinheiro, Olga Ferreira de Souza

**Affiliations:** Electrophysiology Department, Rede D'Or São Luiz, R. Santo Amaro, 80 - Glória, Rio de Janeiro - RJ, 22211-230, Brazil; Clementino Fraga Filho University Hospital, Federal University of Rio de Janeiro, R. Prof. Rodolpho Paulo Rocco, 255 - Cidade Universitária, Rio de Janeiro - RJ, 21941-617, Brazil; Electrophysiology Department, Rede D'Or São Luiz, R. Santo Amaro, 80 - Glória, Rio de Janeiro - RJ, 22211-230, Brazil; Electrophysiology Department, Rede D'Or São Luiz, R. Santo Amaro, 80 - Glória, Rio de Janeiro - RJ, 22211-230, Brazil; Electrophysiology Department, Rede D'Or São Luiz, R. Santo Amaro, 80 - Glória, Rio de Janeiro - RJ, 22211-230, Brazil; Electrophysiology Department, Rede D'Or São Luiz, R. Santo Amaro, 80 - Glória, Rio de Janeiro - RJ, 22211-230, Brazil

**Keywords:** Congenital anomaly, Left atrial appendage, Atrial fibrillation, Electrophysiology, Anticoagulation, Case report

## Abstract

**Background:**

The congenital absence of the left atrial appendage (LAA) is an extremely rare anatomical anomaly, with only 23 cases documented in medical literature. The LAA plays a critical role in thrombus formation, particularly in patients with atrial fibrillation (AF), thus impacting stroke prevention strategies and the management of anticoagulation.

**Case summary:**

We report a 48-year-old male with a 2-year history of hypertension and prior episodes of tachycardic palpitations, who presented with AF and chest pain. During catheter ablation for AF, a transoesophageal echocardiogram revealed the absence of the LAA, initially suspected to be a thrombus. The procedure was suspended until a computed tomography confirmed the congenital absence of the LAA. The patient successfully underwent pulmonary vein isolation with radiofrequency and, after a shared decision-making process, discontinued anticoagulation. He remains asymptomatic without AF recurrence.

**Discussion:**

The congenital absence of the LAA raises important considerations for managing stroke risk in AF patients, as standard thromboembolic risk scores do not account for such anatomical variations. Despite the LAA being the most common site for thrombus formation, stroke risk in AF results from multiple factors beyond a single site of thrombus formation. Left atrial appendage occlusion is a viable alternative for stroke prevention in patients contraindicated for anticoagulation. Recent guidelines recommend individualized risk assessment using scores such as CHA_2_DS_2_-VA, even in atypical cases, ensuring that management strategies are tailored to each patient’s risk profile.

**Learning points**

- Importance of detailed anatomical assessment: The incidental discovery of congenital absence of the left atrial appendage (LAA) during atrial fibrillation ablation, a condition with only 23 reported cases, emphasizes the critical role of comprehensive pre-procedural imaging.
- Personalized anticoagulation strategy: The absence of the LAA challenges traditional anticoagulation practices, as it eliminates the most common site of thrombus formation. According to the 2024 European guidelines, stroke prevention in atrial fibrillation should be individualized, considering that thromboembolic risk is multifactorial. This case demonstrates the need for a tailored anticoagulation approach, especially in patients with lower CHA_2_DS_2_-VA scores and unique anatomical features.

## Introduction

The congenital absence of the left atrial appendage (LAA) is an exceedingly rare anatomical anomaly with sparse documentation in medical literature. At the time of writing, only 23 cases have been documented globally, reflecting the rarity of this condition.^1^ The LAA, though possessing an ambiguous physiological role, is a significant structure in the clinical management of patients with atrial fibrillation (AF). This is primarily due to its involvement in the formation of thrombi, which can precipitate cerebrovascular events.^2^ As such, the LAA is frequently evaluated in patients undergoing cardioversion or ablation for AF to ascertain the absence of thrombi, which would permit the continuation of rhythm control strategies without the risk of embolism.^3^

The absence of specific guidelines on managing anticoagulation in patients with congenital absence of the LAA post-ablation presents a clinical dilemma. The conventional approach to stroke prevention in AF largely relies on the presence of the LAA, given its propensity for thrombus formation. The unexpected discovery of a missing LAA during an AF ablation procedure thus prompts a reconsideration of established protocols and opens a discourse on optimal management strategies for these patients.^4^

In this report, we present the case of a 48-year-old male who underwent an AF ablation, during which the absence of the LAA was incidentally discovered. This case underscores the importance of thorough pre-procedural imaging and raises questions about the need for tailored anticoagulation strategies in such unique anatomical presentations.

## Summary figure

**Figure:**
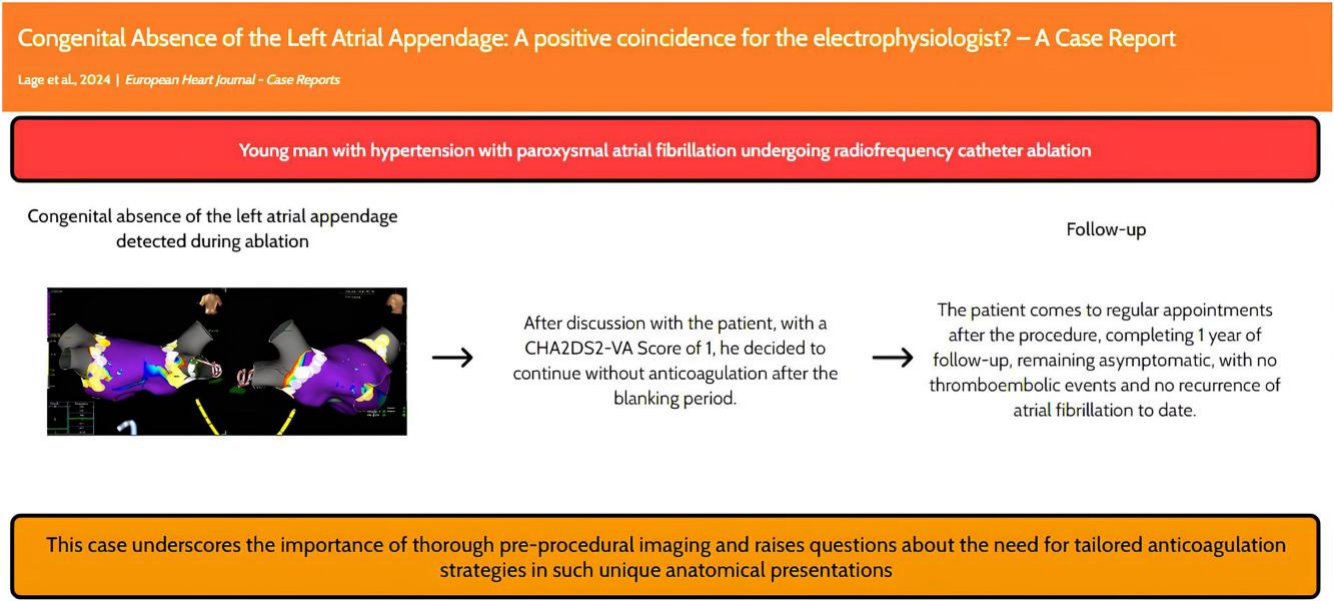


## Case presentation

**Figure 1:**
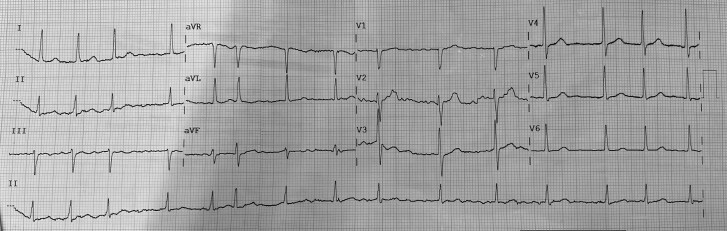
Electrocardiogram of the patient in the cardiointensive unit.

This case involves a 48-year-old overweight male patient with a 2-year history of hypertension, who had not been taking antihypertensive medication regularly despite medical prescriptions, with CHA_2_DS_2_-VA score of 1. He had experienced episodes of tachycardic palpitations in 2023, which prompted him to seek care at other emergency services, where, according to the patient, intravenous medication was administered, and he was discharged shortly thereafter. He had been taking bisoprolol 2.5 mg once daily, propafenone 150 mg twice daily, and rivaroxaban 20 mg once daily irregularly. After a new episode, the patient presented to the Emergency Department of Hospital Glória D'Or, where he was found to have AF with a high ventricular response (*Figure 1*), associated with burning chest pain, and was admitted for better stratification of the chest pain and heart rate control.

During his presentation in the emergency department, he had a blood pressure of 164 × 86 mmHg, a heart rate ranging from 90 to 110 b.p.m., a respiratory rate of 22 irpm, and a cardiovascular physical examination that was normal except for irregular cardiac rhythm. Complementary tests, including laboratory evaluation and chest radiography, revealed no abnormalities, with all results within normal limits, including serum electrolytes.

**Figure 2:**
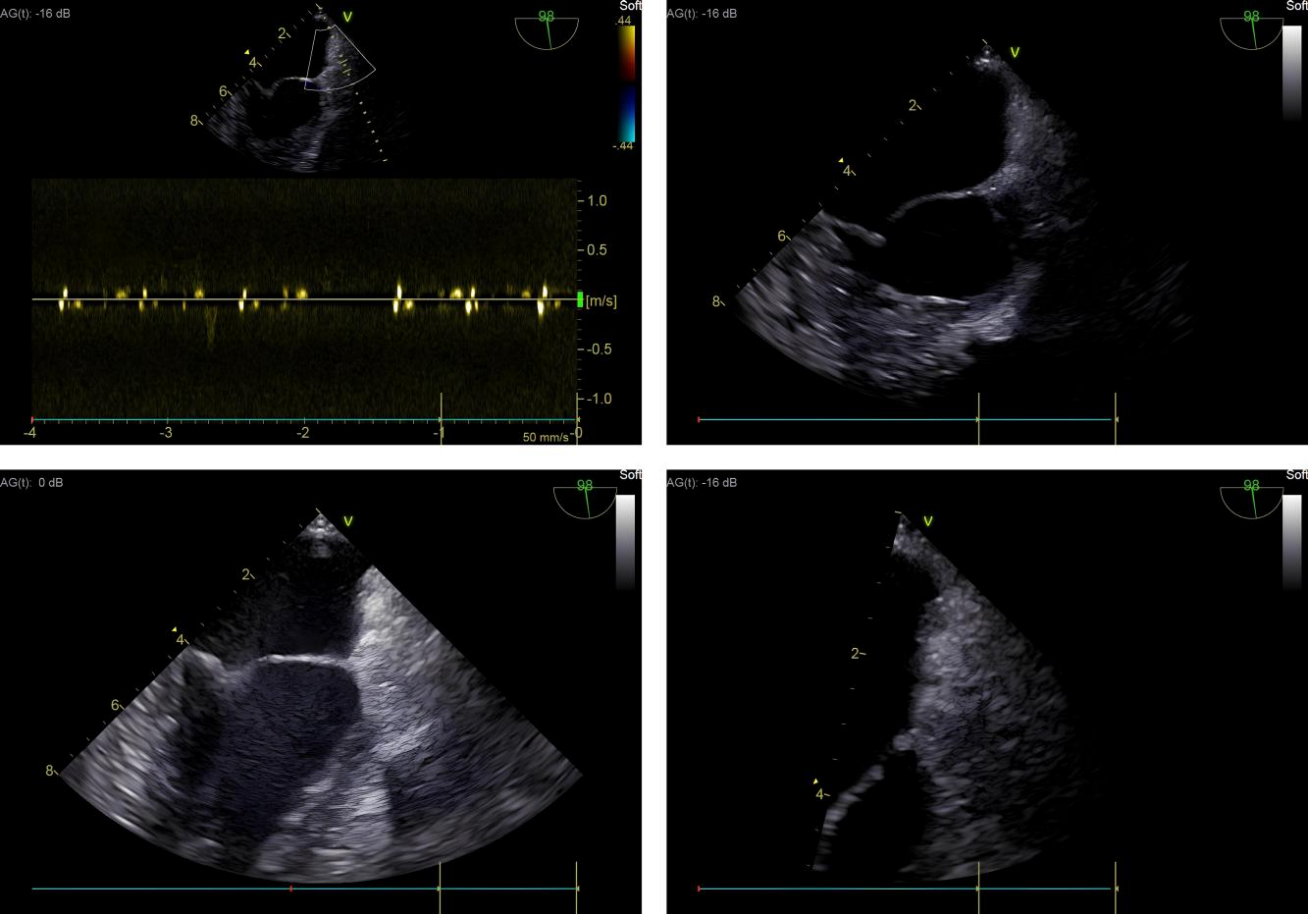
Intraoperative transoesophageal echocardiography with no Doppler flow in the region where the left atrial appendage would be located.

**Figure 3:**
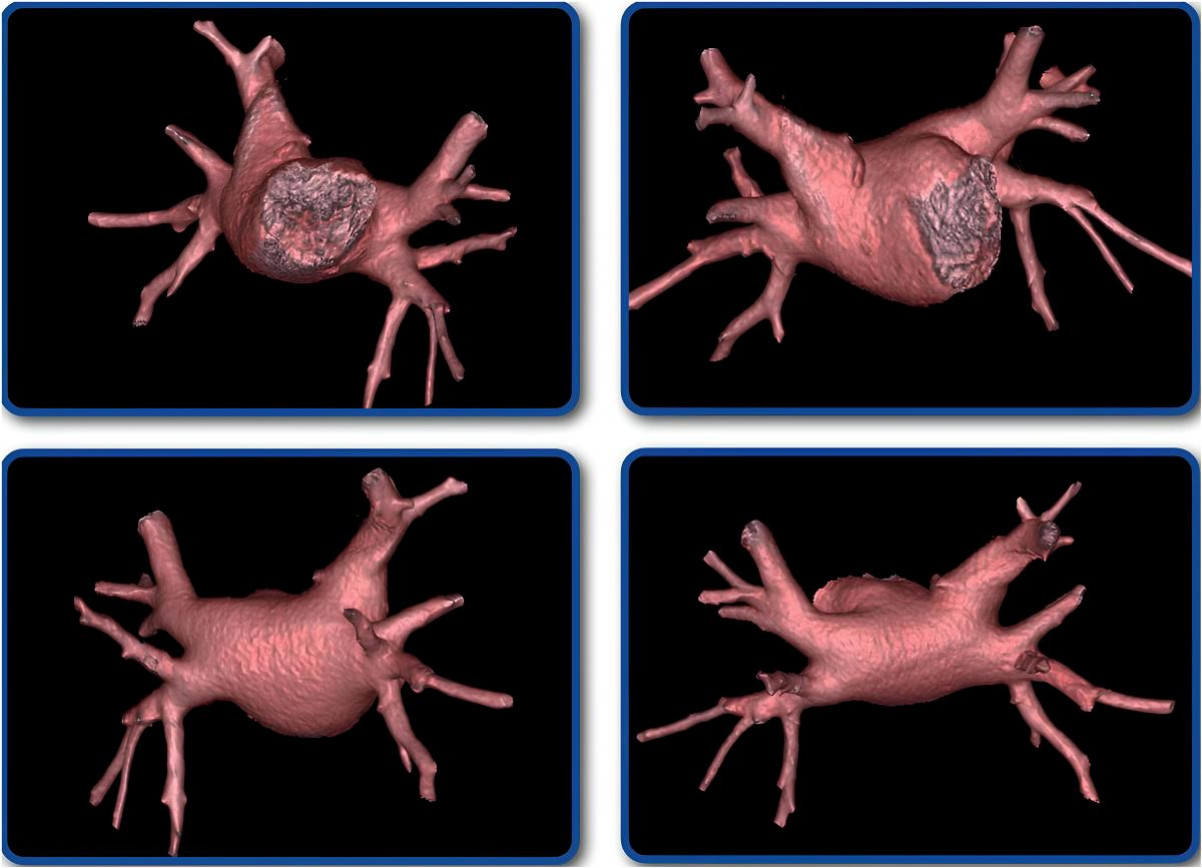
Angio-computed tomography of the left atrium demonstrating the absence of the left atrial appendage.

During hospitalization, the patient was referred for catheter ablation of AF. However, during the intraoperative transoesophageal echocardiogram (*Figure 2*), the LAA could not be characterized, leading the operator to initially suspect the presence of a thrombus in the structure. Consequently, the procedure was suspended, and the patient was returned to the cardiointensive unit. The following day, a left atrium angio-CT was performed, which confirmed the absence of the LAA (*Figure 3*).

**Figure 4:**
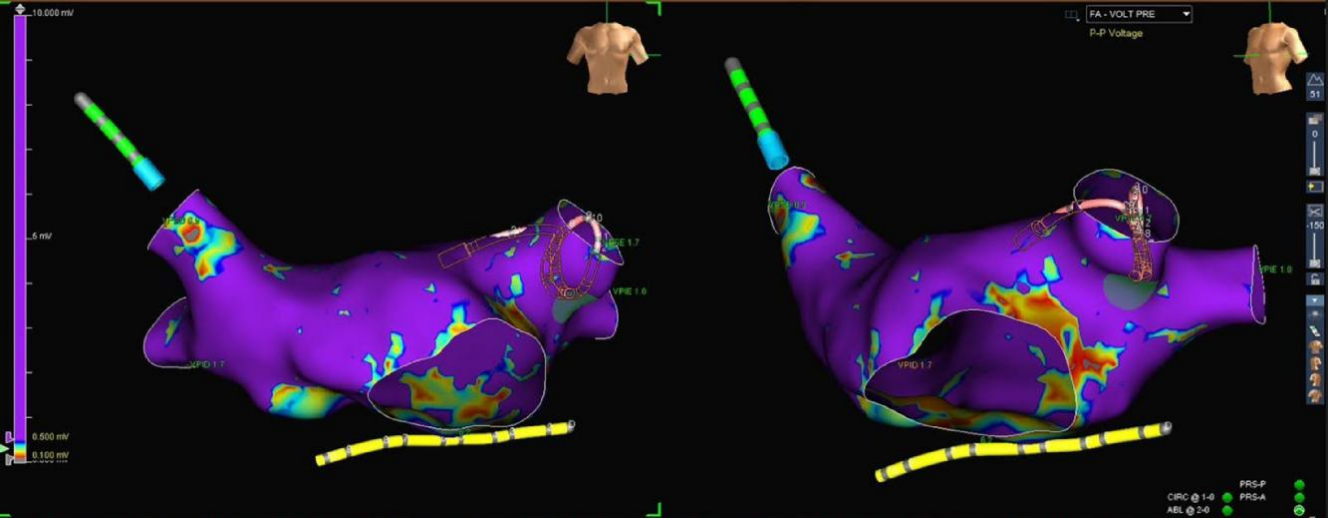
High-density left atrial mapping illustrating the absence of the left atrial appendage.

Thus, the ablation procedure was rescheduled, during which the patient successfully underwent electrical isolation of the four pulmonary veins with radiofrequency. *Figure 4* shows the high-density left atrial mapping, illustrating the absence of the LAA. He remained in the cardiointensive unit for one more day and was subsequently discharged, with the prescription of bisoprolol 2.5 mg once daily, propafenone 150 mg twice daily, valsartan 160 mg once daily, and rivaroxaban 20 mg once daily. After the initial 3-month ‘blanking’ period, anticoagulation was discontinued following a shared decision-making process. After this period, bisoprolol and propafenone were also discontinued. He has remained asymptomatic after a 1-year follow-up and has not experienced any recurrences of AF to date.

## Discussion

The congenital absence of the LAA is a rare finding that poses significant implications for the management of AF, particularly concerning anticoagulation therapy. The LAA is considered the most common site of thrombus formation in patients with AF (without valvular disease), and its presence often dictates the intensity and duration of anticoagulation required.^2^ The congenital absence of this structure, therefore, challenges conventional paradigms.

From a pathophysiological perspective, the LAA is believed to contribute to atrial mechanical function, albeit its exact role remains debated. Its absence may theoretically reduce the risk of thromboembolic events due to the lack of a primary site for thrombus formation.^1^ However, there is insufficient evidence to substantiate this theory fully, necessitating cautious clinical judgment.^3^

In the few reported cases of congenital LAA absence, management has largely been empirical, often extrapolating from guidelines designed for the general AF population. Some clinicians might consider reduced or even no anticoagulation in these patients, arguing that the risk of thrombus formation is inherently lower.^4^ However, the absence of long-term data on stroke outcomes in these patients urges caution. Continuing anticoagulation, albeit potentially at a lower intensity, may be a prudent approach until more definitive evidence becomes available.^2^

Moreover, the role of imaging in detecting congenital LAA absence cannot be overstated. In this case, the discovery was made through a contrast-enhanced computed tomography (CT) scan. The routine use of advanced imaging techniques in patients scheduled for AF ablation could uncover such anomalies pre-procedurally, allowing for better-informed management decisions.^3^

The clinical implications of this case extend beyond the immediate concerns of stroke prevention. For instance, the absence of the LAA may impact the efficacy of catheter ablation, as the appendage’s electrical properties are sometimes targeted during the procedure. This necessitates a tailored approach to ablation in such patients, possibly altering the procedural strategy to account for the unique atrial anatomy.^4^

It is important to note that isolated occlusion of the LAA (a condition possibly analogous to our case) is not yet formally recommended by current guidelines and should only be considered in cases where anticoagulation is contraindicated or when the patient experiences a thromboembolic event despite proper anticoagulation. Even in those patients who undergo cardiac surgery and undergo surgical occlusion of the LAA, anticoagulation must be maintained if the patient’s individual risk is high according to the CHA_2_DS_2_-VA score, therefore being an additional procedure and not a replacement.^5^

Atrial cardiomyopathy is now recognized as an independent risk factor for thromboembolic events, regardless of the presence of AF. This condition is often associated with multimorbidity, leading to higher CHA_2_DS_2_-VASc scores in affected patients. In our case, where the patient only has a risk factor of hypertension, the impact of significant atrial cardiomyopathy is likely lower, as inferred from the left atrial voltage map presented.

## Conclusion

In conclusion, the congenital absence of the LAA is a rare anatomical variant with significant implications for the management of AF. This case highlights the need for further research into the optimal management strategies for such patients, particularly concerning anticoagulation. Until more data are available, a cautious approach, integrating comprehensive imaging and a personalized anticoagulation strategy, is advisable.

## Data Availability

The data that support the findings of this study are available from the corresponding author upon reasonable request.

## Lead author biography

João Gabriel Batista Lage, MD, PhD, is a cardiologist specializing in cardiac arrhythmias. He completed his medical degree at Fluminense Federal University (UFF), where he received the Academic Laureate Award. Dr Lage trained in Cardiology at the Heart Institute (InCor) of the University of São Paulo Medical School (FMUSP), where he also earned his PhD in Cardiology with a focus on arrhythmias. He is a member of the European Society of Cardiology (ESC) and the European Heart Rhythm Association (EHRA). Currently, he practices at the Clementino Fraga Filho University Hospital - Federal University of Rio de Janeiro (UFRJ) and Rede D'Or São Luiz in Rio de Janeiro, Brazil.
